# Neural and hormonal mechanisms of appetite regulation during eating

**DOI:** 10.3389/fnut.2025.1484827

**Published:** 2025-03-24

**Authors:** Xurui Sun, Binghan Liu, Yuan Yuan, Ying Rong, Rui Pang, Qiu Li

**Affiliations:** ^1^Key Laboratory of Endocrine Glucose and Lipids Metabolism and Brain Aging, Ministry of Education; Department of Endocrinology, Shandong Provincial Hospital Affiliated to Shandong First Medical University, Jinan, China; ^2^Department of Neurosurgery, Shandong Provincial Hospital Affiliated to Shandong First Medical University, Jinan, China; ^3^Department of Clinical Nutrition, Shandong Provincial Hospital Affiliated to Shandong First Medical University, Jinan, China

**Keywords:** appetite control, neural pathways, blood nutrient level, dietary behavior, vagal afferent nerves

## Abstract

Numerous animal and clinical studies have demonstrated that the arcuate nucleus of the hypothalamus, a central regulator of appetite, plays a significant role in modulating feeding behavior. However, current research primarily focuses on long-term dietary changes and their effects on the body, with limited investigation into neuroendocrine dynamics during individual meals across diverse populations. In contrast to long-term dietary adjustments, directives for dietary behavior during a specific meal are more actionable, potentially enhancing patient adherence and achieving better outcomes in dietary behavior interventions. This review aimed to explore the neural pathways and endocrine changes activated by gastrointestinal expansion and variations in blood nutrient levels during a single meal, with the goal of informing dietary behavior guidance.

## 1 Introduction

The driving forces of eating can be attributed to two distinct factors: (1) the sensation of hunger caused by gastrointestinal peristalsis and (2) signals from the brain that generate the desire to eat. The hypothalamic arcuate nucleus (ARC), located adjacent to the third ventricle, is widely recognized as a central regulator of appetite. This region features a specialized and more permeable blood–brain barrier compared to other brain regions (1). The increased permeability allows circulating nutrients and hormones—such as glucose, leptin, and insulin (3)—to directly access agouti-related protein (AgRP) and pro-opiomelanocortin (POMC) neurons within the ARC. This direct access makes these neurons highly responsive to nutrient fluctuations, facilitating the regulation of energy homeostasis. Various appetite-regulating hormones including insulin, leptin, and peptide YY (PYY), as well as small-molecule nutrients such as amino acids and glucose, can directly stimulate these neurons and play a crucial role in appetite regulation.

Eating habits have been shown to affect energy metabolism and contribute to the development of metabolic diseases. Specifically, faster eating (4), higher cooking temperatures (5), fewer chews (6), and late eating (7) have been associated with increased food intake and increased risk of obesity, diabetes, and hypertension. A high-fat diet (HFD) can remodel brain neurons (8), alter the dominant species of intestinal flora (9), increase body fat deposition, and contribute to metabolic disorders (10). Conversely, intermittent fasting improves insulin sensitivity, reduces blood triglyceride and cholesterol levels, alleviates chronic inflammation (11), and helps regulate metabolic disorders (12).

To date, few studies have focused on the effects of feeding during a specific meal or conducted a comprehensive analysis of neurological and humoral changes. Compared to existing dietary guidelines, behavior guidance for a single meal is more specific and practical, aiding patients in self-control and implementation. In this review, we explore the factors that influence appetite, focusing on the feeding process during a complete eating cycle. We analyze the immediate neurological and humoral changes observed in animals. Due to limitations in current research, this article predominantly focuses on experimental mice, while findings from mouse studies may offer insights applicable to human research. We also discuss how these changes impact appetite during this process. This review provides insights for dietary behavior guidance.

## 2 Changes in neurohumoral regulation during eating

For a relatively balanced meal in terms of both quantity and quality, gastric emptying in humans typically takes 4–6 h. Feelings of hunger typically emerge around this time, marking the starting point of the feeding process addressed in this review. The orexigenic effect of AgRP depends on the release of neuropeptide Y (NPY) and gamma-aminobutyric acid (GABA) (2, 13). Mouse experiments have demonstrated that mice deficient in NPY or GABA transporters do not rapidly increase food intake when AgRP neurons are activated. This finding indicates that NPY and GABA mediate the rapid, short-term effects of AgRP on feeding behavior (2, 14). In contrast, the orexigenic effect induced by AgRP itself is slower and more prolonged (2). In addition to stimulating appetite, activating AgRP decreases energy expenditure, enhances carbohydrate utilization, and reduces fat breakdown (2, 15). Under conditions of extreme hunger, the AgRP→parabrachial nucleus (PBN) pathway suppresses pain responses, allowing animals to prioritize foraging for food (16). Mouse experiments have demonstrated that activating AgRP/NPY neurons increases their willingness to take greater risks when seeking food (17, 18).

**Figure 1:**
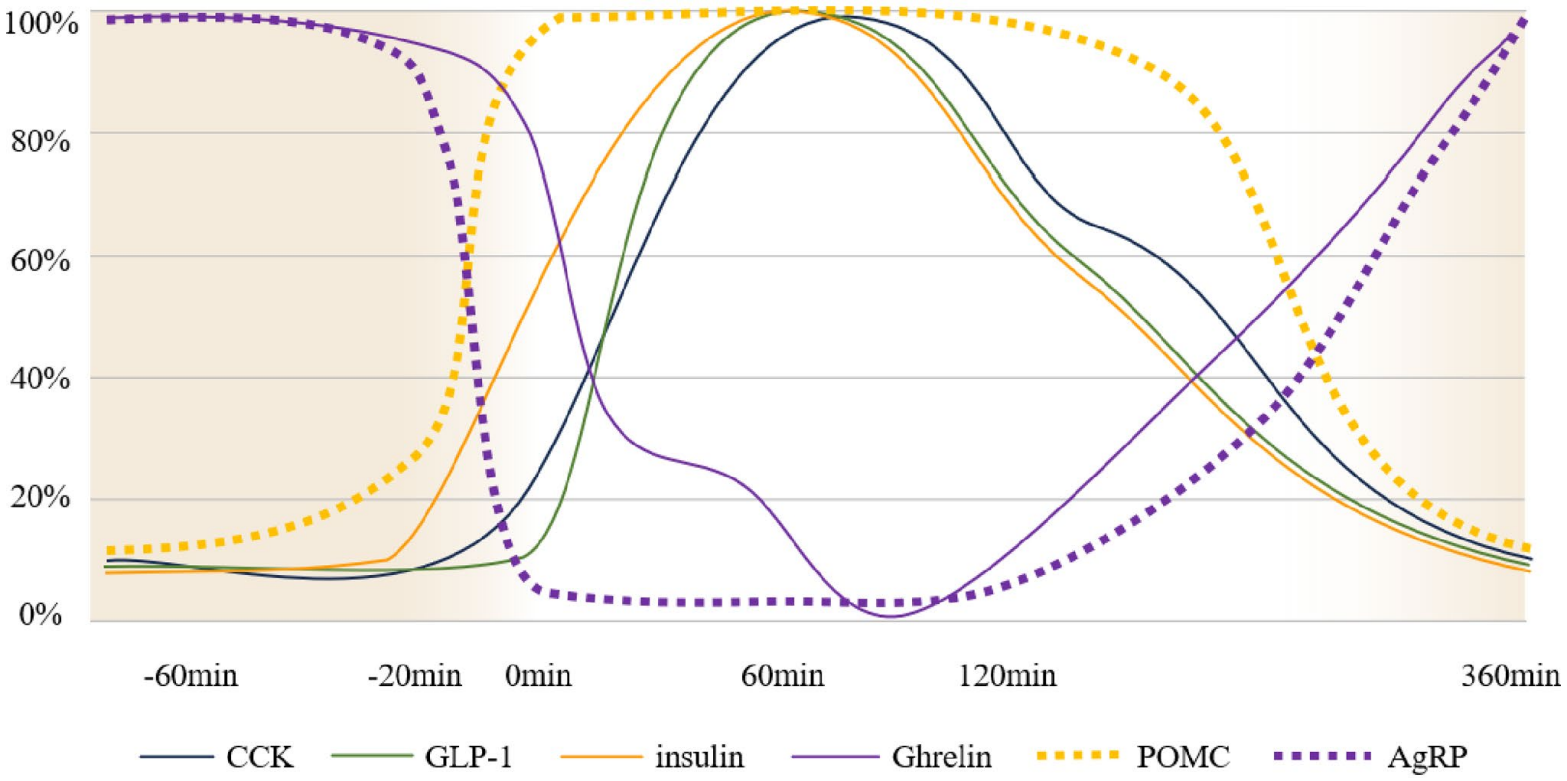
Schematic diagram of neurohumoral changes over time. The vertical axis represents the percentage of normalized changes in neural activity and hormone levels (109–114), 100% consider it as complete activation of nerves/peak release of hormones in the body, 0% consider it as complete inhibition of nerves/hormone content in the body measured as 0. The horizontal axis indicates meal time. The timeline assumes a state of hunger at −60 min, food discovery at 20 min, and the initiation of meals at 0 min. Gastric emptying concludes at 360 min, at which point the system returns to a hunger state. It should be noted that this is only a schematic diagram and does not represent, for example, an equal release of GLP-1 and insulin during the eating process.

Upon food discovery, visual (19) and olfactory (20) signals transmitted into the brain activate different brain areas; AgRP secretion rapidly decreases, while POMC neurons, which antagonize AgRP, are rapidly activated. Simultaneously, saliva, digestive enzymes, insulin, and other substances are secreted to prepare food intake (21). Vagal receptors in the oropharyngeal area detect changes in taste and tension, activating the vagus nerve to initiate the vagovagal reflex of the stomach and facilitate the smooth entry of food into the stomach. Vagal receptors distributed throughout the gastrointestinal tract sense changes in tension and nutrient levels within the digestive system. These stimuli are transmitted to the brain, inducing satiety and activating secretory cells in the intestine to release appetite-suppressing hormones, such as PYY and serotonin, which stimulate the hypothalamus to create a feeling of fullness (22). Through the coordinated actions of multiple factors, the eating event concludes as food is gradually digested into chyme in the stomach. As nutrients are absorbed by intestinal epithelial cells, blood glucose levels rise, providing the energy required for growth and activity. Over time, these nutrients—primarily glucose—are utilized, leading to decline in blood glucose levels, reduction in insulin secretion, and gradual increase in AgRP/NPY neuron activity, eventually triggering the next sensation of hunger. The trend of changes in the main neurohumoral regulation during eating is illustrated in Figure 1.

### 2.1 Before swallowing

During the period between detection of food signals and swallowing, the body undergoes a series of anticipatory responses, collectively referred to as cephalic phase responses. These responses include the secretion of saliva, digestive enzymes, and insulin (23); they ensure rapid digestion, efficient food absorption, and effective nutrient metabolism (24). Recently, cephalic phase responses have gained increasing attention. Studies on rodents and humans have shown that, when transmitted to corresponding areas of the cerebral cortex, sensory food stimuli, such as visual (25), taste (26), and olfactory cues (27), have corresponding effects on subsequent eating behavior through different pathways, some of which are associated with the reward system.

The nucleus tractus solitarius (NTS) integrates these signals and relays them to the dorsal motor nucleus of the vagus nerve (DMV), initiating vagal nerve activity and mediating the early secretion of insulin (28, 29). Although this hypothesis remains unconfirmed, it is well established that cephalic phase insulin release positively correlates with the amount of food ingested during a meal. The cephalic phase insulin release also counteracts hepatic glucose production mediated by glucagon, helping to maintain relatively stable blood glucose levels (23).

**Figure 2:**
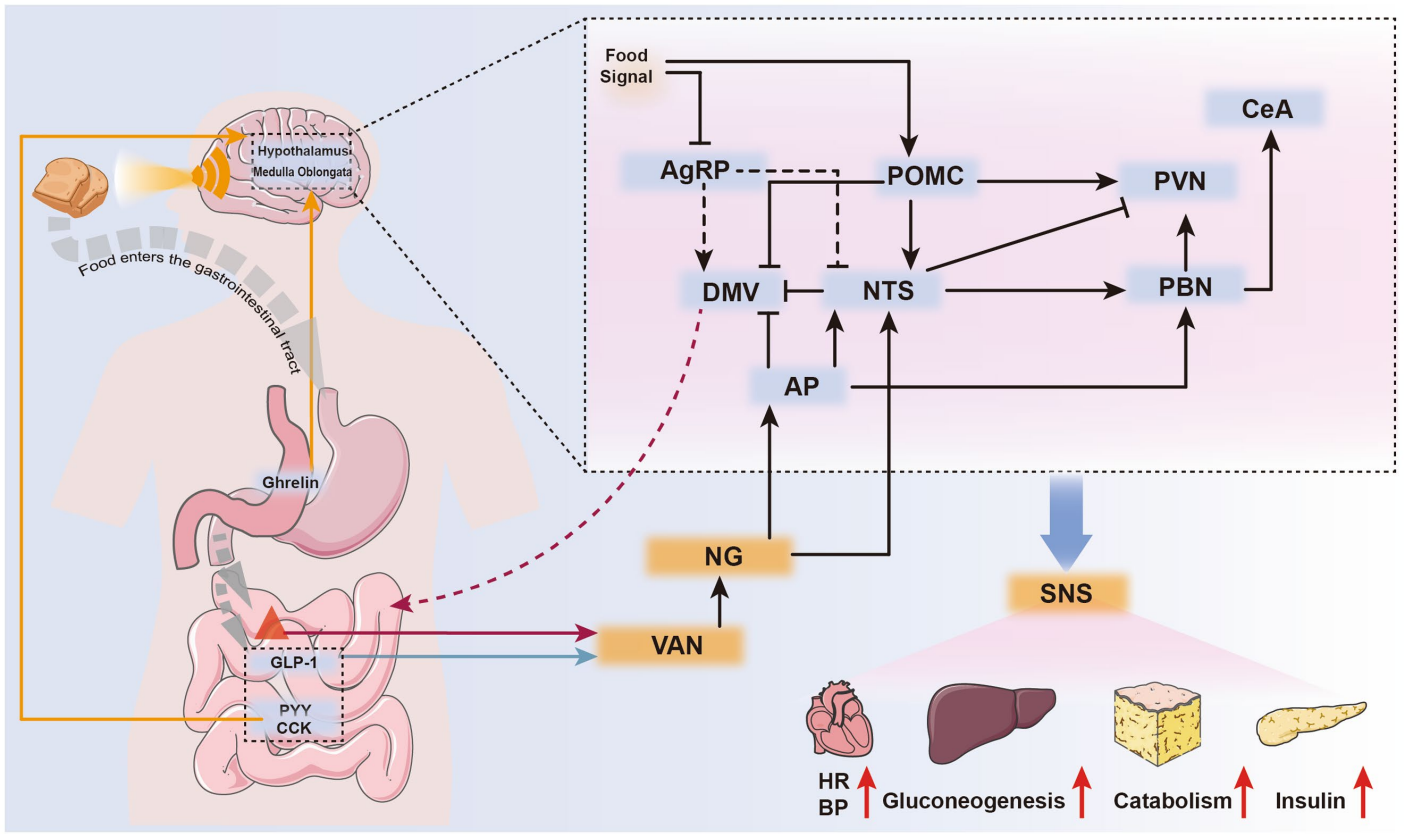
Neurohumoral pathways mediating feeding cessation during eating. Upon sensing food signals, the body can immediately suppress AgRP neurons and activate POMC neurons, preparing for food intake. Ghrelin, secreted by gastric wall cells during hunger, is currently the only known gastrointestinal hormone with appetite-stimulating effects. It can directly stimulate specific brain nuclei through the bloodstream or exert indirect effects via vagal sensory receptors (orange arrows).After eating, gastrointestinal hormones such as PYY and CCK inhibit appetite by directly stimulating relevant brain nuclei through the humoral circulation (orange arrows).Gastrointestinal hormones released after eating, along with mechanical stimuli from gastrointestinal distension, are sensed by different vagal sensory receptors. These stimuli are transmitted to the corresponding brain nuclei, generating a sense of satiety (satiety) and controlling gastrointestinal motility to create a feeling of fullness (satiation) (black arrows). The parabrachial nucleus (PBN), upon receiving these stimuli, activates the central amygdala (CeA) in the cerebral cortex, mediating the sensation of satiety. Activated POMC, NTS, PVN, and AP neurons can stimulate the sympathetic nervous system, leading to systemic responses such as increased heart rate, elevated blood pressure, enhanced hepatic gluconeogenesis, accelerated fat metabolism, and increased insulin secretion.

POMC neurons also influence the liver’s regulation of early-phase blood glucose after a meal. Upon sensing food cues, POMC neurons are rapidly activated and regulate the sympathetic nervous system of the liver in an mTOR-dependent manner, thereby controlling hepatic glycogenolysis (30). Additionally, AgRP neurons are rapidly and uniformly inhibited when detecting food cues, with the level of inhibition positively correlated with palatability and expected energy content of the food (31) (Figure 2). This inhibitory response is mediated by GABAergic neurons expressing leptin receptors in the dorsomedial hypothalamus (DMH) (24), and its duration is proportional to energy expenditure (31).

Although both types of neurons respond rapidly to food cues, they do not directly mediate the initiation or termination of eating behaviors. The dorsal vagal complex, located in the brainstem, receives input signals from the hypothalamus and integrates them with signals from the gastrointestinal tract via the vagus nerve, playing a key role in mediating eating behavior (32).

### 2.2 Commencement of eating

Food enters the stomach after chewing and swallowing, mixes with gastric juice, breaks down into chyme and then passes into the intestine for absorption. This process involves complex neurohumoral changes, primarily mediated by the sensory branches of the vagus nerve distributed throughout the stomach and intestinal walls. These changes stimulate vagal receptors in the oropharyngeal region, which transmit feeding signals to the stomach via the vagovagal reflex, initiating gastric accommodation. Gastric accommodation maintains the relative stability of intragastric pressure (IGP), a factor significantly correlated with satiety. A brief decrease in IGP is observed at the onset of eating (33). As food accumulates in the stomach, the muscles of the gastric cardia, fundus, and body gradually become tense, increasing IGP. The tension receptors widely distributed in the gastric walls are stimulated, transmitting signals via the nodose ganglion (NG) to the NTS, which mediates the sensation of fullness (34). The rate of gastric emptying during eating is higher than usual, allowing some incompletely digested chyme to enter the intestine, where it stimulates tension and chemoreceptors in the duodenal wall (32). Additionally, tension receptors in the gastrointestinal wall may be involved in regulating insulin secretion during eating (35).

Several gastrointestinal hormones, including ghrelin, glucagon-like peptide-1 (GLP-1), PYY, serotonin, and cholecystokinin (CCK), are involved in appetite regulation. Ghrelin, secreted by P/D1 cells in the gastric fundus, increases during fasting and rapidly decreases after eating (31), promoting feeding by stimulating AgRP neurons. Enteroendocrine cells in the duodenum detect changes in intestinal tension and nutrient levels, releasing hormones such as serotonin and CCK. These hormones activate vagal afferents or enter the bloodstream, mediating the sensation of satiety (32).

### 2.3 Factors influencing eating cessation

As the amount of food entering the stomach increases, the tension in the gastrointestinal wall also rises, causing a gradual increase in IGP and the onset of satiation. Vagal afferent nerves (VANs) detect this increased tension and transmit the signals to the NTS. Beyond its role in regulating gastrointestinal motility via the dorsal motor nucleus of the vagus (36), NTS Calcr neurons mediate the cessation of feeding by suppressing AgRP activity and activating non-calcitonin gene-related peptide (CGRP) neurons in the PBN (37). Additionally, satiety hormones stimulate neurons such as POMC to create a sense of fullness (38). Aversive signals, discomfort (39), and other negative feedback mechanisms also contribute to the cessation of feeding. These mechanisms are primarily mediated by the area postrema (AP) and PBN (see Section 3.2 for details). The detailed process of cessation of eating behavior is shown in Figure 2.

## 3 Changes in the vagus nerve

The vagus nerve, also known as the tenth cranial nerve, originates in the medulla oblongata of the brainstem. It consists of four types of fibers—somatic sensory, somatic motor, visceral sensory, and visceral motor— and extensively innervates the gastrointestinal tract. The vagus nerve detects changes in gastrointestinal tension and hormone levels and transmits these signals to the NTS. Subsequently, impulses are generated to modulate the feeding process.

### 3.1 Visceral sensory afferent fibers

Different subtypes of VANs are distributed across various organs and are specialized to perceive distinct stimuli. Five subtypes are predominant in the gastrointestinal tract: calcitonin gene-related peptide 1 (Calca), GLP-1 receptor (GLP-1R), G protein-coupled receptor 65 (Gpr65), vasoactive intestinal peptide (Vip), and oxytocin receptor (Oxtr) (24).

The termination of feeding behavior in mice is triggered primarily by gastric distension rather than an increase in internal nutrients (40), indicating that mechanosensory fibers play a dominant role in this process. VAN^GLP-1R+^ fibers are distributed in the gastric wall, and VAN^Oxtr+^ fibers are found in the intestinal wall. Both serve as mechanosensory fibers and possess intraganglionic laminar endings (22, 41–43). Acute chemical and optogenetic stimulation of these two fiber types in mice induces the cessation of feeding behavior (24, 41, 42) and suppresses the activity of AgRP neurons. Notably, stimulation of VAN^GLP-1R+^ fibers results in strong but transient inhibition, while stimulation of VAN^Oxtr+^ fibers causes prolonged suppression of AgRP neurons and reduces their responsiveness to food cues (42). Additionally, the activation of VAN^GLP-1R+^ fibers enhances glucose uptake by skeletal muscles and improves glucose tolerance (41).

Intraperitoneal administration of CCK and GLP-1, which are released by intestinal endocrine cells (44) into the mouse intestine, reduces food intake. Forced inhibition of VAN^GLP-1R+^ fibers alleviates the feeding reduction mediated by CCK but does not affect appetite suppression induced by GLP-1R agonists, such as liraglutide. Furthermore, CCK receptors have been detected on VAN^GLP-1R+^ fibers (41), suggesting that CCK, rather than GLP-1, is the hormone that directly activates these fibers.

In the gastric mucosa, VAN^Calca+^, and at the tips of intestinal villi, VAN^Gpr65+^ and VAN^Vip+^ fibers primarily sense changes in gastrointestinal hormones and nutrients, thereby regulating glucose metabolism (22, 41). Activation of VAN^Gpr65+^ fibers increases the expression of phosphoenolpyruvate carboxykinase, a key enzyme in gluconeogenesis, enhancing hepatic gluconeogenesis and elevating blood glucose levels (41, 45). However, its effects on appetite regulation have not yet been determined (24).

### 3.2 Afferent ganglia and NTS

The cell bodies of the sensory neurons of the vagus nerve are located in two nodose ganglia near the base of the skull, close to the carotid artery (43). These ganglia serve as crucial sites for mediating satiety signals. Their central axons project to the dorsal hindbrain, activating neurons in the NTS. The NTS processes mechanical signals from the gastrointestinal tract and chemical signals, such as CCK, GLP-1, PYY, and serotonin, which are secreted by enteroendocrine cells (43, 46, 47). Using Ca^2+^ flux measurements, Williams et al. (22) demonstrated that different subtypes of neurons in the NG can sense gastrointestinal tension and hormonal signals independently. NG^GLP-1R+^ cells can sense tension signals from the stomach and parts of the intestine, transmit these signals to the NTS and AP, and subsequently inhibit the activation of AgRP neurons (42). Neurons sensing hormonal signals express Gpr65, which, similar to NG^GLP-1R+^, activates the NTS and AP and modulates IGP (42). A study by Han et al. (48) demonstrated that the activation of the right vagus nerve (NG) in mice induces sustained self-stimulation behavior, similar to the behaviors observed when cortical reward centers are activated, and promotes dopamine release in the substantia nigra. In contrast, the activation of the left NG did not elicit similar responses. This suggests that the activation of the gastrointestinal vagus nerve may indirectly regulate feeding behavior by stimulating cortical reward centers (48).

Signals from the intestine are transmitted via the NG to the dorsal vagal complex in the brainstem, which includes the NTS, AP, and DMV. In the dorsal vagal complex, these signals are integrated and transmitted to the hypothalamus, PBN, and other regions to regulate appetite while controlling gastrointestinal motility through the DMV (49, 50). The AP, located outside the blood–brain barrier, primarily mediates aversive signals and receives a small portion of stimuli from VANs (34). Hormones involved in satiety, such as CCK and PYY, can activate the AP (41, 51–53) and subsequently the NTS. The NTS plays a critical regulatory role in food intake and energy metabolism by processing mechanical and chemical signals from the gastrointestinal tract. Cheng et al. (37) reported that the activation of the NTS suppresses the expression of AgRP/NPY neurons in the ARC, thereby inhibiting feeding. Additionally, the NTS mediates the cessation of feeding by activating downstream regions, including the PBN, paraventricular nucleus of the hypothalamus, and DMH (37). The PBN, a small cluster of nuclei located at the midbrain-pons junction, acts as a relay center for visceral and gustatory signals as well as somatosensory information, including pain, temperature, and itch. The PBN receives inhibitory inputs from AgRP neurons (54) and excitatory signals from the NTS (55), allowing it to regulate discomfort and energy metabolism. By activating the central nucleus of the amygdala, the PBN suppresses appetite (56). Activation of Calcr-expressing neurons in the NTS (NTSCalcr neurons) transmits signals to the PBN, specifically activating PBN non-CGRP neurons, leading to feeding suppression.

Furthermore, Cheng et al. (37) demonstrated that silencing NTSCalcr neurons in mice resulted in chronic increases in food intake and subsequent weight gain (37). Additionally, the activation of NTS CCK neurons triggers CGRP neurons in the PBN. When NTS CCK neurons detect toxic substances in the blood (e.g., LiCl), the discomfort signals they mediate can outweigh hunger signals driven by AgRP neurons. In such cases, PBN CGRP neurons relay these aversive signals to the central amygdala, resulting in appetite suppression (53).

### 3.3 Damage to the vagus nerve caused by HFD

Short-term acute HFD intake activates the reward system on the edge of the middle brain, and the dopamine energy neurons participating in the reward system can be projected into the regulation of dietary behavior (57). However, impairment of satiety signals induced by long-term HFD is widely recognized as a primary contributor to obesity. HFD not only reduces the sensitivity of VAN^GLP-1R/CCK^ neurons (58) but also affects the expression of GLP-1R and CCK in neurons (59). This reduction subsequently decreases the expression of POMC and cocaine- and amphetamine-regulated transcript (60), thereby weakening satiety signals. CCK receptors on VAN^CCK^ neurons are coexpressed with leptin receptors (61). Damage to VAN^CCK^ can lead to reduced leptin receptor expression, resulting in leptin resistance and increased appetite (62). The gut microbiota mediates *CCK* gene expression by producing short-chain fatty acids (63), which can directly activate VANs (64). Disruption of the gut microbiota by HFD reduces the abundance of short-chain fatty acid-producing bacteria, such as lactobacilli, and decreases the diversity of short-chain fatty acids. This disruption may lead to increased microglial activity in the NTS, generating neuroinflammation, promoting synaptic remodeling, and impairing satiety signal transmission (58). Rodents with obesity induced by HFD exhibit reduced activation of postprandial brainstem neurons (65), specifically a decrease in NTS neuron activation (66).

In summary, HFD detrimentally affects multiple processes involved in satiety signal generation and transmission mediated by the vagus nerve. These effects are primarily driven by changes in CCK, leptin, and gut microbiota. Further research is needed to determine if acute HFD intake affects dietary behavior through other mechanisms.

## 4 Changes in the sympathetic nervous system

Although the sympathetic nervous system is generally believed to be inhibited during feeding behavior, recent studies suggest that sympathetic excitation also plays a role in both the initiation and cessation of feeding behavior. Evidence indicates that sympathetic excitation occurs before the cessation of feeding (67). Neuropeptides within the melanocortin system involved in the regulation of satiety, such as adrenocorticotropic hormone, α-melanocyte-stimulating hormone (MSH), β-MSH, and γ-MSH, exhibit varying excitatory effects on the sympathetic nervous system. Conversely, neurons expressing AgRP/NPY, which project to the paraventricular nucleus (PVN) and DMH, suppress sympathetic activity by releasing NPY (68). Hormones involved in dietary metabolism, such as leptin and insulin, regulate sympathetic nerve activity either directly or indirectly.

### 4.1 Melanocortin system regulates sympathetic nerve activity

As previously mentioned, the gradual increase in gastrointestinal hormones such as insulin, PYY, and CCK, as well as increasing gastrointestinal tension during a meal, is transmitted to the NTS. This activation stimulates POMC neurons in the ARC, generating a sensation of satiety and mediating the cessation of feeding. POMC neurons release various neuropeptides, including adrenocorticotropic hormone, α-MSH, β-MSH, γ-MSH, and β-endorphin, which bind to different melanocortin receptor subtypes (MC1R-MC5R) to produce distinct effects. Among these, the α-MSH neuropeptide binds to MC4R and plays a central role in regulating appetite, energy balance, and sympathetic nervous system activity (69). The activation of MC4R-expressing sympathetic preganglionic neurons in the DMV, NTS, and intermediolateral cell column has been shown to restore sympathetic activity in mice and enhance the thermogenic response mediated by MC4R (70). Mice with MC4R deficiency exhibit significantly reduced sympathetic activity compared to wild-type mice when consuming an HFD. Rahmouni et al. revealed that the MC4R pathway is essential for the modulation of insulin and leptin effects on renal sympathetic nerve activity (71). Hypertension typically associated with obesity through sympathetic activation is not observed in diet-induced obese rats with MC4R deficiency (72). Additionally, studies have shown that MC4R-deficient mice lose the ability to convert white adipose tissue into brown adipose tissue in response to cold exposure, resulting in significantly reduced thermogenic capacity (73). These findings indicate that MC4R enhances sympathetic nervous system activity to increase energy expenditure, thereby maintaining metabolic stability.

In addition, the role of MC4R in regulating blood pressure is significant and should not be overlooked. Intracerebroventricular injection of α-MSH in awake rats increased central venous pressure and heart rate within 1 h, and they returned to baseline levels after 2 h, which may be associated with sympathetic activation (74). However, the effect of α-MSH on blood pressure was completely abolished in animals with MC4R knockout (75). Direct injection of the MC4R agonist melanotan II into the PVN increases renal sympathetic excitability and central venous pressure. However, this effect is not observed in PVN pretreated with MC4R antagonists, such as AgRP (76).

### 4.2 Effect of NPY on sympathetic nerve activity

NPY can inhibit KCl-activated POMC neurons in a dose-dependent manner, thereby reducing sympathetic nervous system activity (77). Furthermore, the inhibitory effects of NPY on the sympathetic nervous system are primarily observed in the PVN. The suppression of brown adipose tissue thermogenesis (78) and the promotion of white adipose tissue and liver lipogenesis (79, 80) by NPY may be linked to its suppression of sympathetic nervous system activity. Additionally, the reduction in vascular smooth muscle sympathetic nerve activity induced by NPY facilitates increased blood flow to the gastrointestinal tract and liver, thereby enhancing digestion and absorption (81). NPY receptors are also present in the DMH, where NPY can inhibit sympathetic nerve activity, leading to a reduction in mean arterial pressure and heart rate (37, 82–84). Notably, blocking NPY1 receptors (NPY1Rs) in both the DMH and PVN resulted in differential effects: blocking NPY1Rs in the DMH completely reversed the inhibitory effect of AgRP/NPY neurons on sympathetic nerve activity, whereas blocking NPY1Rs in the PVN did not. Activation of the PVN and DMH elicited comparable effects on the sympathetic nerve activity, mean arterial pressure, and heart rate. The DMH is known to innervate and stimulate the PVN, which likely accounts for the observed similarity in responses (83).

Preliminary experiments have demonstrated the anticipatory nature of AgRP/NPY neurons, whose activity rapidly declines prior to feeding. The extent of this decline correlates with the anticipated energy density of the food to be consumed (37). The rapid reduction in NPY levels increases inhibition of the sympathetic nervous system, potentially contributing to the increase in heart rate and blood pressure during feeding. Additionally, the increase in gastrointestinal blood flow activates baroreceptors, leading to vasodilation to maintain stable blood flow and facilitate the post-feeding absorption process.

### 4.3 Hormones regulate sympathetic nerve activity

Leptin- and insulin-induced activation of the sympathetic nervous system is closely associated with maintaining homeostasis. Leptin receptors, which are widely distributed throughout the hypothalamus, respond to leptin stimulation and converge signals in the PVN. These signals subsequently activate the rostral ventrolateral medulla, which excites sympathetic preganglionic neurons in the spinal cord (85, 86).

Studies have shown that the effect of leptin on the sympathetic nervous system is sex-dependent. In female rats, the sympathetic nervous system’s response to leptin varies cyclically with fluctuations in estrogen levels (87). Unlike in male rats, leptin-induced excitation of the sympathetic nervous system in female rats does not increase blood pressure (87, 88). Additionally, female rats with higher estrogen levels are reportedly more sensitive to the anorectic effects of leptin and less sensitive to those of insulin (89). The differential effects of leptin between sexes are not attributed to differences in sites or pathways of action but rather to a positive interaction between leptin and estrogen at the cellular level (90, 91).

Unlike leptin, the effect of insulin on sympathetic activation is not sex-dependent. In rats, insulin primarily increases the sympathetic excitability of peripheral vessels, with the most pronounced effect on spinal sympathetic neurons. Additionally, insulin enhances the excitability of human muscle sympathetic nerves and increases the muscle vascular baroreceptor reflex (92). A meta-analysis by Grassi et al. (93), involving 314 individuals with diabetes and healthy controls across 11 studies, confirmed that patients with type 2 diabetes exhibit higher sympathetic excitability than healthy individuals and those with type 1 diabetes. Patients with type 2 diabetes showed significantly elevated muscle sympathetic nerve activity, which was associated with plasma insulin levels, corroborating the excitatory effect of insulin on the sympathetic nervous system.

### 4.4 Other factors influencing sympathetic nervous system activity

#### 4.4.1 Impact of dietary components on sympathetic nervous system activity

Postprandial activation of the sympathetic nervous system has been observed, with different dietary components influencing its activation to varying degrees. High carbohydrate intake is associated with greater sympathetic activation (94). However, the statistical significance of these changes remains unclear (95). Postprandial insulin secretion promotes peripheral vasodilation, enhancing nutrient absorption. This is followed by a decrease in venous return, which stimulates the sympathetic nervous system. In some older individuals, postprandial hypotension may occur due to reduced sympathetic activity (96). Reducing dietary carbohydrate intake may help alleviate postprandial hypotension in these cases.

#### 4.4.2 Interaction between obesity and sympathetic nervous system activity

Obesity is believed to increase sympathetic activity. Research suggests that obesity decreases NPY content or mRNA levels in the ARC and PVN (97–99). This reduction enhances sympathetic activation during eating, which may explain why obese individuals tend to eat faster than others. HFD has been shown to reduce NPY1R expression in the DMH in males but not in females (100, 101). Reduced NPY release or receptor levels may contribute to the increased sympathetic activity associated with obesity (81). Sakamoto et al. demonstrated that overnutrition leads to excessive activation of the sympathetic nervous system, resulting in insulin resistance and metabolic disorders, ultimately contributing to obesity.

Reducing catecholamine release from the sympathetic nervous system has been shown to protect the body from the adverse effects of overnutrition-induced insulin resistance, hyperinsulinemia, hyperglucagonemia, adipose tissue dysfunction, and fatty liver disease (102).

## 5 Summary and future directions

Numerous studies have investigated the mechanisms underlying appetite generation and satiation, the effects of diet on appetite, and the role of dietary modifications in controlling appetite. Due to space limitations, certain factors regulating appetite, such as sleep (103–105), sex (106), alcohol (107), and stress (108), were not thoroughly reviewed in this paper. Most of these studies have primarily focused on the nutritional components of the diet and the long-term effects of dietary control, with relatively few examining specific behaviors associated with a single meal. The advent of optogenetics has enabled precise stimulation and observation of neurons, resulting in increased research on the neural pathways that regulate dietary behavior. This review summarizes existing gut-brain neural pathways and provides a comprehensive description of neurohumoral changes throughout the entire eating process, from the onset of hunger, discovery of food, and initiation of eating to the gradual feeling of satiety, cessation of eating, and completion of gastric emptying. It also analyzes changes in appetite during this process, aiming to inspire future research on dietary regulation. It is anticipated that future dietary guidance will place greater emphasis on specific meal processes to enhance patient compliance. Future studies should prioritize investigating how factors such as eating speed and nutritional content influence satiety from a meal-specific perspective. Additionally, personalized assessments of neural and endocrine alterations during the eating process across different individuals are essential to provide more precise dietary recommendations and achieve the goals of behavioral eating counseling.

## Glossary

AgRP: Agouti-related protein
AP: Area postrema
ARC: Arcuate nucleus
CCK: Cholecystokinin
CGRP: Calcitonin gene-related peptide
DMH: Dorsomedial hypothalamus
DMV: Dorsal motor nucleus of the vagus
GABA: Gamma-aminobutyric acid
GLP-1: Glucagon-like peptide-1
GLP-1R: Glucagon-like peptide-1 receptor
Gpr65: G protein-coupled receptor 65
HFD: High-fat diet
IGP: Intragastric pressure
MC1R-MC5R: Melanocortin receptor 1–5
MSH: Melanocyte-stimulating hormone
NG: Nodose ganglion
NPY: Neuropeptide Y
NTS: Nucleus of the solitary tract
Oxtr: Oxytocin receptor
PBN: Parabrachial nucleus
POMC: Pro-opiomelanocortin
PVN: Paraventricular nucleus
PYY: Peptide YY
VANs: Vagal afferent nerves
Vip: Vasoactive intestinal peptide
